# Autologous humanized PDX modeling for immuno-oncology recapitulates features of the human tumor microenvironment

**DOI:** 10.1136/jitc-2023-006921

**Published:** 2023-07-24

**Authors:** Michael Chiorazzi, Jan Martinek, Bradley Krasnick, Yunjiang Zheng, Keenan J Robbins, Rihao Qu, Gabriel Kaufmann, Zachary Skidmore, Melani Juric, Laura A Henze, Frederic Brösecke, Adam Adonyi, Jun Zhao, Liang Shan, Esen Sefik, Jacqueline Mudd, Ye Bi, S Peter Goedegebuure, Malachi Griffith, Obi Griffith, Abimbola Oyedeji, Sofia Fertuzinhos, Rolando Garcia-Milian, Daniel Boffa, Frank Detterbeck, Andrew Dhanasopon, Justin Blasberg, Benjamin Judson, Scott Gettinger, Katerina Politi, Yuval Kluger, Karolina Palucka, Ryan C Fields, Richard A Flavell

**Affiliations:** 1 Department of Internal Medicine, Section of Medical Oncology, Yale School of Medicine, New Haven, Connecticut, USA; 2 Department of Immunobiology, Yale School of Medicine, New Haven, Connecticut, USA; 3 Jackson Laboratory - Farmington, Farmington, Connecticut, USA; 4 Alvin J Siteman Cancer Center, St Louis, Missouri, USA; 5 Department of Surgery, Washington University School of Medicine in Saint Louis, St Louis, Missouri, USA; 6 Department of Pathology, Yale School of Medicine, New Haven, Connecticut, USA; 7 Bioinformatics Support Program, Cushing/Whitney Medical Library, Yale School of Medicine, New Haven, Connecticut, USA; 8 Department of Surgery, Section of Thoracic Oncology, Yale School of Medicine, New Haven, Connecticut, USA; 9 Department of Surgery, Section of Otolaryngology, Yale School of Medicine, New Haven, Connecticut, USA; 10 Howard Hughes Medical Institute, New York, New York, USA

**Keywords:** Immunity, Innate, Immunotherapy, Inflammation, Macrophages, Tumor Microenvironment

## Abstract

**Background:**

Interactions between immune and tumor cells are critical to determining cancer progression and response. In addition, preclinical prediction of immune-related drug efficacy is limited by interspecies differences between human and mouse, as well as inter-person germline and somatic variation. To address these gaps, we developed an autologous system that models the tumor microenvironment (TME) from individual patients with solid tumors.

**Method:**

With patient-derived bone marrow hematopoietic stem and progenitor cells (HSPCs), we engrafted a patient’s hematopoietic system in MISTRG6 mice, followed by transfer of patient-derived xenograft (PDX) tissue, providing a fully genetically matched model to recapitulate the individual’s TME. We used this system to prospectively study tumor-immune interactions in patients with solid tumor.

**Results:**

Autologous PDX mice generated innate and adaptive immune populations; these cells populated the TME; and tumors from autologously engrafted mice grew larger than tumors from non-engrafted littermate controls. Single-cell transcriptomics revealed a prominent vascular endothelial growth factor A (VEGFA) signature in TME myeloid cells, and inhibition of human VEGF-A abrogated enhanced growth.

**Conclusions:**

Humanization of the interleukin 6 locus in MISTRG6 mice enhances HSPC engraftment, making it feasible to model tumor-immune interactions in an autologous manner from a bedside bone marrow aspirate. The TME from these autologous tumors display hallmarks of the human TME including innate and adaptive immune activation and provide a platform for preclinical drug testing.

**WHAT IS ALREADY KNOWN ON THIS TOPIC**

- Studies using humanized mice for cancer investigation have been limited by low efficiency of engraftment of human hematopoietic stem and progenitor cells (HSPCs), requiring large numbers of patient-derived, or unrelated donor HSPCs to reconstitute a human immune system.

**WHAT THIS STUDY ADDS**

- The MISTRG6 system supports human hematopoietic reconstitution with enhanced efficiency, making it possible to repopulate mice with the immune system of solid tumor patients through bone marrow aspiration. Subsequent transplantation of autologous tumor tissue provides a fully genetically matched system to study innate and adaptive immune influence on the tumor microenvironment (TME). These autologous models encompass a patient’s tumor and host genetic makeup and allow for preclinical testing of drugs on human cells.

**HOW THIS STUDY MIGHT AFFECT RESEARCH, PRACTICE OR POLICY**

- These studies demonstrate the utility of next generation humanized mouse models for investigating the biology of the TME, especially the impact of human innate immune cells, as well as testing therapies that modulate the human tumor niche.

The immune milieu within tumors, consisting of diverse cell types including adaptive immune cells as well as macrophages, dendritic cells, natural killer and other innate immune cells, is critical to determining cancer outcome, be it progression or regression.^1^ Macrophages especially can have pro-growth and anti-growth properties within the tumor microenvironment (TME) in various cancers.^2–4^ However, the immune TME has been challenging to model, owing to inherent interspecies differences.^5–9^ While advances in humanized mice have expanded the repertoire of human immune cells that can repopulate immunodeficient mice, the hematopoietic stem and progenitor cells (HSPCs) used for transplantation have been largely limited to those derived from fetal or neonatal stem cell donors. As a result, these experiments are mismatched between HSPC donor and tumor tissue, creating allogeneic immune responses as well as assaying the immune function of the unrelated HSPC donor. The ability to preclinically model an individual adult patient with cancer, capturing the unique features of an individual such as germline genetic determinants of immunity and somatic tumor heterogeneity, is critical to advancing our understanding of interpersonal differences in tumor progression and response to cancer therapies.

MISTRG mice, in which the **M**-CSF (CSF1), CSF2/**I**L3, **S**IRPA, **T**HPO genes were humanized on a **R**AG2^−/−^, IL2R**G** ^−/−^ immunodeficient background, generate functional human monocytes, tissue macrophages, alveolar macrophages, and natural killer (NK) cells in a profile more similar to humans than other models, affording an opportunity to model multiple human immune cell types that interact with tumor cells in a human TME.^10–12^ However, efficiency of engraftment in most mouse strains and even the relatively efficient MISTRG mouse precludes the routine engraftment of adult HSPCs. Here, we show that the MISTRG6^13^ strain is a much more adept host recipient of human HSPCs and demonstrate the utility of this system to study the TME in adult patients with solid tumor by modeling tumor-immune interactions in a fully genetically matched, autologous manner. We found that humanization of the IL-6 locus significantly improved human hematopoietic engraftment of MISTRG6 mice compared with prior models, allowing efficient modeling of individual patients’ tumor-immune interactions using low numbers of HSPCs obtained prospectively from bone marrow (BM) aspirates. Patient-derived xenograft (PDX) tumors grown in mice engrafted with autologous CD34^+^ HSPCs displayed infiltration of patient immune cells of both innate (eg, macrophage) and adaptive (eg, T cell) immune subtypes. The transcriptional signatures of the latter imply activation and exhaustion in the TME, whereas the signatures of the former suggest protumor production of vascular endothelial growth factor A (VEGF-A) in the TME as a major determinant of PDX progression in this model.

## Results

### MISTRG6 displays enhanced proportions of human hematopoietic cells, including innate immune cell types

**Figure 1:**
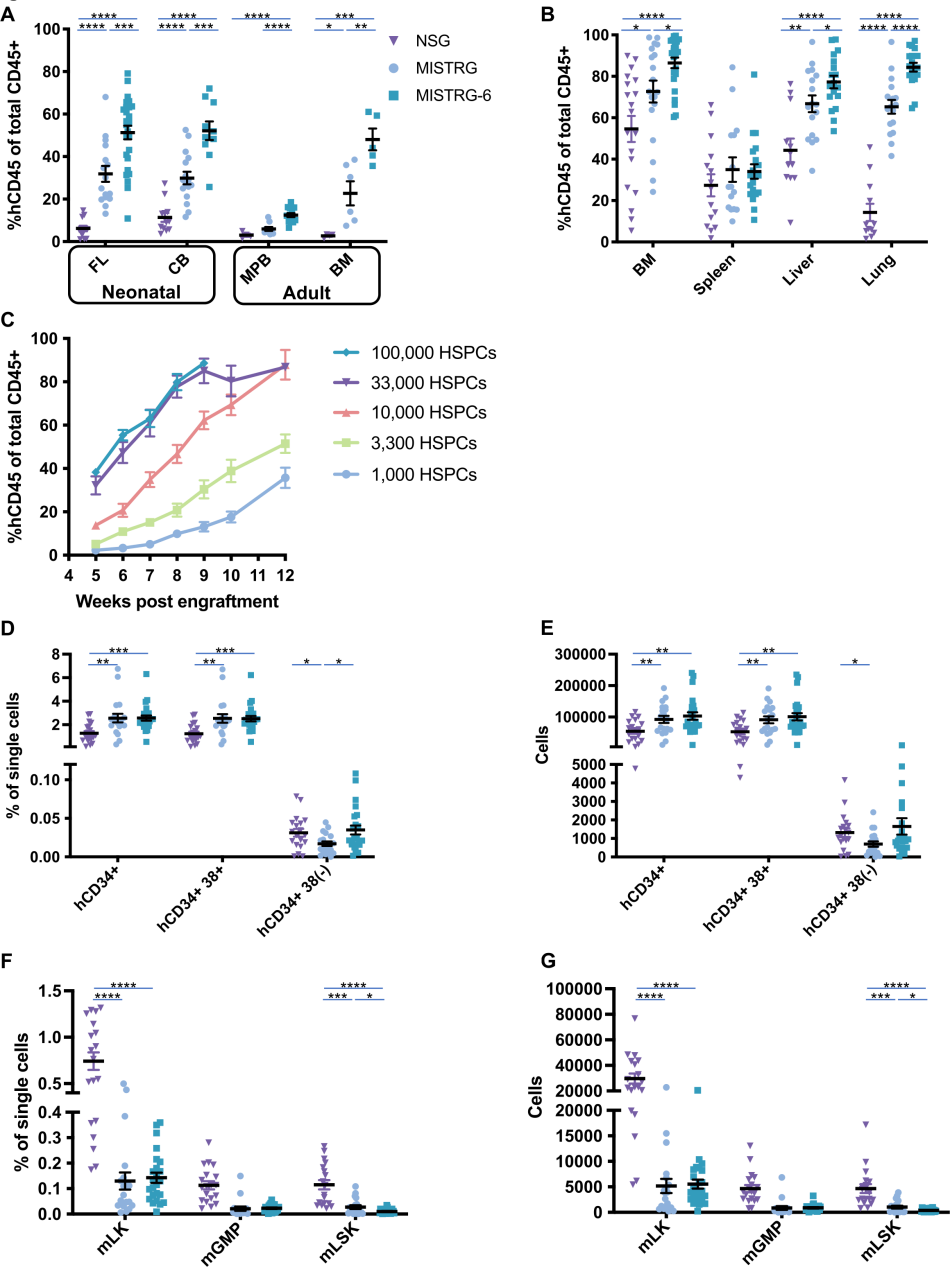
Humanization of the IL-6 locus enhances human hematopoietic engraftment in MISTRG6 mice. (A) Human hematopoietic engraftment (percent of hCD45^+^ cells as a proportion of total hCD45^+^ and mCD45^+^ cells) in peripheral blood of NSG (triangles), MISTRG (circles) and MISTRG6 (squares) mice engrafted with equal numbers of CD34^+^ HSPCs from indicated sources; *p<0.05, **p<0.01, ***p<0.001, ****p<0.0001, unpaired parametric t-test; bars indicate mean and SEM); each dot represents a single mouse. 1–3 day-old pups were irradiated with 150 rads, intrahepatically injected with 100,000 FL, 50,000 CB, or 100,000–180,000 adult PB CD34^+^ cells. Blood engraftment was measured at 5–7 weeks post engraftment. (B) Human hematopoietic engraftment (%hCD45^+^ of total CD45^+^) in indicated tissues of NSG, MISTRG and MISTRG6 mice engrafted with equal numbers of CD34^+^ HSPCs. (C) Longitudinal analysis of human CD45^+^ cells in peripheral blood of MISTRG6 mice engrafted with varying HSPC numbers as indicated; n=6–11 for each group. (D) Percentage among single cells of hCD34^+^, hCD34^+^hCD38^+^, and hCD34^+^hCD38^(−)^ cells detected in BM of NSG, MISTRG and MISTRG6 mice engrafted with equal numbers of hCD34^+^ HSPCs. (E) Absolute numbers of cells in BM of mice in (D). (F) Percentage among single cells of mouse mLK, mGMP and mLSK cells detected by flow cytometry in BM of NSG, MISTRG and MISTRG6 mice engrafted with equal numbers of CD34^+^ HSPCs. (G.) Absolute numbers of cells from BM of mice in (F). BM, bone marrow; CB, cord blood; FL, fetal liver; HSPCs, hematopoietic stem and progenitor cells; IL, interleukin; MPB, mobilized peripheral blood; PB, peripheral blood.

Given the divergence between human and mouse interleukin (IL)-6 protein (41% identity) and to improve support of human HSPCs, we used the MISTRG6 mouse that bears the human IL-6 gene in place of the murine gene in the same chromosomal location, thereby preserving the surrounding regulatory sequences for IL-6 control.^13 14^ When intrahepatically engrafted with equivalent numbers of CD34^+^ cells from human fetal liver (FL), neonatal cord blood, adult mobilized peripheral blood (MPB), or adult BM, MISTRG6 mice harbored greatly increased human hematopoietic cells as a proportion of total hematopoietic cells in peripheral blood compared with NOD-scid-gamma (NSG) and MISTRG mice (figure 1A). Levels of human hematopoietic cells were also higher in most tissues, including BM, liver, and lung (figure 1B), with the greatest differences being between MISTRG6 and NSG hosts. No significant difference was detected in spleen across strains (figure 1B). Moreover, we found that MISTRG6 mice could be engrafted with as few as 1000 human HSPCs and achieve robust hematopoietic transplantation after 10–12 weeks (figure 1C), indicating the efficiency of this strain in supporting the growth of hematopoietic cells.

To better elucidate the mechanism responsible for this enhanced human engraftment, we enumerated human and mouse hematopoietic progenitors in BM of NSG, MISTRG, and MISTRG6 mice. This revealed that human progenitors, including CD34^+^ and CD34^+^CD38^+^ cells, were significantly increased in both frequency and absolute numbers in MISTRG and MISTRG6 mice compared with NSG mice (figure 1D–E), and that the mouse hematopoietic lin^(−)^cKit^+^ (mLK) and lin^(−)^Sca1^+^cKit^+^ (mLSK) progenitor populations were significantly diminished, while granulomonocytic progenitor (mGMP) cells were not significantly different (figure 1F–G).^15^ In addition, MISTRG6 mice displayed more human hCD34^+^CD38^(-)^ and fewer mLSK cells compared with MISTRG. Collectively, these findings suggest that the enhanced hematopoietic engraftment observed in MISTRG6 is, in part, a consequence of increased human progenitor frequency and reduced mouse progenitor competition.

Humanization of the CSF1 locus in MISTRG enables development of human myeloid lineage cells (CD33^+^) with robust functionality.^10–12^ As expected, this property was preserved in the MISTRG6 model, regardless of human HSPC source or tissue assayed (online supplemental figure 1A-F). MISTRG6 mice had a significantly increased proportion of hCD33^+^ myeloid cells than NSG mice in peripheral blood when engrafted with FL-derived or MPB-derived CD34^+^ cells, with concomitant decrease in frequency of hCD19^+^ B cells (online supplemental figure 1A, B). In addition, when engrafted with MPB-derived CD34^+^ cells, hNKp46^+^ NK cells were present in equal proportions in MISTRG and MISTRG6 mice but were lacking in NSG mice (online supplemental figure 1B). These trends persisted in tissues, with CD33^+^ myeloid cells being significantly increased in MISTRG and MISTRG6 mice in spleen, liver, and lung (online supplemental figure 1C–F). In BM, spleen, liver, and lung, MISTRG6 mice had significantly larger proportions of human NK cells and hCD66b^+^SSC^hi^ granulocytes as well as fewer B cells than NSG mice. T-cell proportions did not differ significantly between the strains (online supplemental figure 1C–F).

10.1136/jitc-2023-006921.supp1 Supplementary data

In summary, MISTRG6 is highly efficient at supporting development of human hematopoietic cells, including innate immune cells.

### MISTRG6 allows efficient engraftment of patient-derived HSPCs

**Figure 2:**
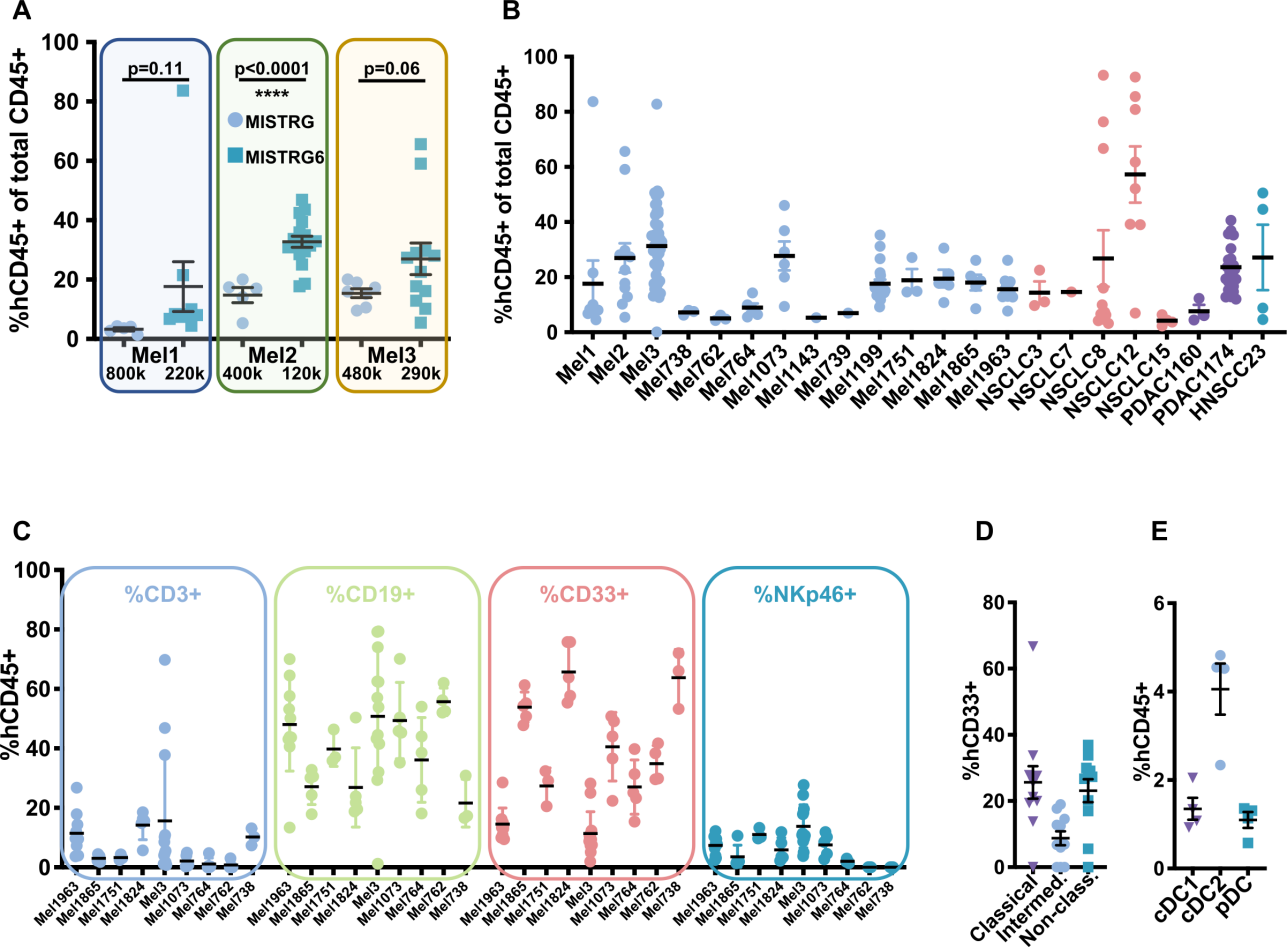
Improved engraftment in MISTRG6 compared with MISTRG mice of HSPCs from patients with solid tumor, with human innate and adaptive immune cells represented. (A) Analysis at 8 weeks of age of peripheral blood of MISTRG (circles) and MISTRG6 (squares) mice engrafted with HSPCs from the indicated patients with melanoma with the HSPC dose shown below; note improved human engraftment levels despite fewer HSPCs introduced in MISTRG6 compared with MISTRG. (B) Human hematopoietic engraftment of MISTRG6 mice with 100–250 k HSPCs derived from adult patients with melanoma (Mel), non-small cell lung cancer (NSCLC), pancreatic adenocarcinoma (PDAC), and squamous cell carcinoma of the head and neck (HNSCC); each dot represents a single mouse. (C) Percentage of human T cells (CD3^+^), B cells (CD19^+^), myeloid cells (CD33^+^) and NK cells (NKp46^+^) out of total human hematopoietic cells in peripheral blood of autologously engrafted mice from the indicated patients. (D) Proportions of human monocyte subsets (CD14^+^CD16^(−)^ classical, triangles; CD14^+^CD16^+^ intermediate, circles; CD14^(−)^CD16^+^non-classical, squares) of total CD33^+^ myeloid cells in peripheral blood of Mel1963 autologously engrafted MISTRG6 mice. (E) Proportions of human dendritic cell subsets (HLA-DR^+^CD11c^low^CD141^+^ cDC1, triangles; CD11c^+^CD1c^+^ cDC2, circles; CD14^(−)^CD11c^(−)^CD303^+^ pDC, squares) of total hCD45^+^ cells in spleens of Mel1963 autologously engrafted MISTRG6 mice. HSPCs, hematopoietic stem and progenitor cells; NK, natural killer.

Having documented that MISTRG6 better supports development of human hematopoietic cells from adult donors and requires transplantation of fewer cells to do so, we sought to apply this prospectively to model individual patients’ TME. For proof of concept, we initially used CD34^+^ cells from granulocyte colony stimulating factor (G-CSF)-mobilized peripheral blood samples that were collected from patients with metastatic melanoma enrolled in a dendritic cell vaccine trial.^16^ Consistent with the data in figure 1 using HSPCs from healthy adults, this showed that engrafting fewer cells yielded higher levels of human engraftment in MISTRG6 compared with MISTRG mice, and that efficient engraftment was feasible with 100,000 to 300,000 CD34^+^ cells (figure 2A). Importantly, this enabled engrafting >30 recipient animals. For example, for patient Mel2, engrafting 400,000 CD34^+^ cells in MISTRG hosts yielded a mean of 14.8% human hematopoietic cells in mouse peripheral blood, while engrafting 120,000 CD34^+^ cells in MISTRG6 hosts yielded significantly increased mean human hematopoietic engraftment (34.7%) (figure 2A). Similar results were obtained for patients Mel1 and Mel3, for whom fewer cells achieved greater proportions of human hematopoietic cells in MISTRG6 compared with MISTRG, although not achieving statistical significance (figure 2A). Nevertheless, these results suggest that the enhanced engraftment and growth in MISTRG6 compared with MISTRG is influenced by the individual donor, since Mel3 was superior to Mel1, even though the proportion of HSPCs transferred was greater for Mel1 than Mel3 (800 vs 220 for Mel1 and 480 vs 290 for Mel3).

With successful generation of humanized mice bearing individual patients’ immune systems, we expanded these efforts to prospective collection of BM-derived CD34^+^ cells from patients under active treatment along with tumor tissue from the same patient (ie, an autologous platform). Under Institutional Review Board-approved protocols at two cancer centers, we enrolled patients with melanoma (Mel), non-small cell lung cancer (NSCLC), pancreatic adenocarcinoma (PDAC), and head and neck squamous cell carcinoma (HNSCC) to provide BM aspirate, peripheral blood, and tumor tissue at the time of surgery or biopsy. CD34^+^ cells were isolated from BM aspirates using magnetic bead purification and cryopreserved. Viable tumor tissue was used to generate PDXs in non-engrafted NSG or MISTRG6 hosts. Overall, 71 patients were enrolled, 46 Mel, 19 NSCLC, 4 PDAC, 2 HNSCC, ages 22–85, 39% women (online supplemental table 1). These yielded autologous, immune-reconstituted MISTRG6 hosts from 14 patients with Mel, 5 patients with NSCLC, 2 patients with PDAC, and 1 patient with HNSCC (figure 2B). Of note, we did not observe a correlation between age of patient or pretreatment with HSPC reconstitution (not shown).

Autologously engrafted MISTRG6 mice displayed the gamut of human immune cells of adaptive and innate types in peripheral blood at 7 weeks of age (figure 2C). Notably, this included CD33^+^ myeloid lineage cells such as CD14^+^CD16^−^ classical, CD14^+^CD16^+^ intermediate, and CD14^−^CD16^+^ non-classical monocytes in peripheral blood (figure 2D). Moreover, dendritic cells (DCs), key innate immune cells for initiation of antitumor and other immune responses that derive from CD33^+^ cells,^17^ were readily detected by flow cytometry in spleens of autologously-engrafted mice, including human cDC1, cDC2, and pDC populations (figure 2E).

### MISTRG6 mice bearing a patient’s hematopoietic cells support autologous PDX growth

**Figure 3:**
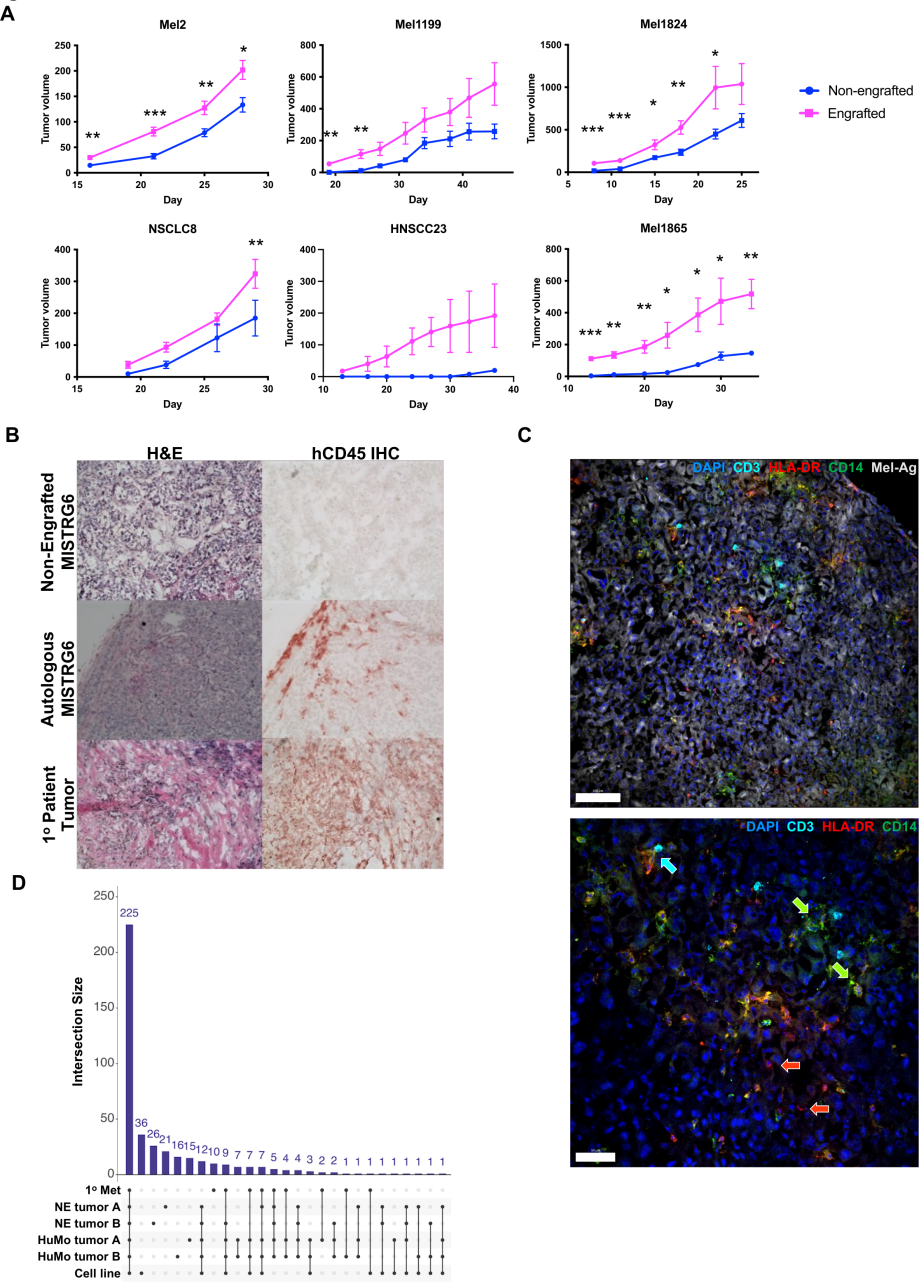
Autologous engraftment enhances PDX growth and human immune cell infiltrate demonstrates tumor-immune interactions. (A) PDX growth in MISTRG6 littermates engrafted (magenta) or non-engrafted (blue) with autologous HSPCs from indicated patients (see online supplemental figure 2 for comprehensive data). (B) H&E (left) and hCD45 immunohistochemical (right) staining of tumors harvested from Mel1073 PDX grown in non-engrafted hosts (top), autologously engrafted MISTRG6 hosts and the patient’s primary tumor (bottom). (C) Sections of Mel1199 PDX tumor grown in autologously engrafted host stained for human infiltrating immune cells; top panel blue=DAPI, cyan=hCD3, green=hCD14, red=hHLA DR, white=melanoma antigen, scale bar=100 um. Bottom panel shows the same markers without melanoma antigen staining. Green arrows highlight HLA-DR^+^CD14^+^ macrophages while red arrows highlight HLA-DR^+^CD14^(−)^ antigen-presenting cells. Cyan arrow highlights T cell-macrophage interaction. Scale bar=50 um. (D.) Number of somatic mutations (compared with germline reference) shared between the indicated samples: Mel738 patient’s surgical resection sample = 1^o^ Met; PDX tumors from non-engrafted mice (lacking human immune cells) = NE tumor A and B; PDX tumors from mice with autologous engraftment=HuMo A and B; cell line derived from the patient’s tumor=cell line. Note that most mutations (225) are shared among all samples. HNSCC, squamous cell carcinoma of the head and neck; HSPCs, hematopoietic stem and progenitor cells; Mel, melanoma; NSCLC, non-small cell lung cancer; PDX, patient-derived xenograft.

Having achieved successful engraftment of patient hematopoietic systems in MISTRG6 hosts, we next subcutaneously introduced the patient’s matched PDX tumor tissue to generate autologously engrafted PDX mice. We monitored tumor growth by caliper measurements in autologously engrafted and non-engrafted (ie, mice lacking human hematopoietic cells) MISTRG6 littermates, finding that autologous PDX experiments were feasible in MISTRG6 with tumors reaching up to 1000 mm^3^ (figure 3A and online supplemental figure 2). As expected, PDXs from different patients displayed distinct tumor growth dynamics, with some PDXs growing more or less rapidly. For most patients, tumors grown in autologous HSPC-engrafted hosts were significantly larger than in non-engrafted hosts (figure 3A and online supplemental figure 3A). Harvested xenografted tumors were histologically similar to the parental version and showed hCD45^+^ hematopoietic infiltration when grown in engrafted hosts; tumors grown in non-engrafted hosts lacked hCD45 staining consistent with these PDXs having been passaged and lacking passenger human immune cells from the patients (figure 3B). Multicolor immunofluorescence staining of PDX tumors demonstrated that human immune cells, including CD3^+^ T cells, CD14^+^ and HLA-DR^+^ myeloid cells, penetrated deeply into the tumor and co-localized with tumor cells as well as with other engrafted immune cells (figure 3C). Indeed, HLA-DR^+^CD14^+^ macrophages and HLA-DR^+^CD14^(−)^ antigen-presenting cells were present, and direct physical interaction between T cells and macrophages was evident (figure 3C, bottom panel).

### Whole-exome sequencing reveals preservation of mutations from patient to humanized xenograft

To determine the mutational landscape of tumors generated in MISTRG6 hosts bearing autologous hematopoietic cells and compare to a patient’s tumor as well as those in PDXs grown in non-engrafted mice, we performed whole-exome sequencing (WES) on these samples from Mel738. Peripheral blood mononuclear cells (PBMCs) from the patient were used as reference. This analysis indicated that 225 somatic changes were shared between the patient’s surgical resection sample (1° Met), two PDX tumors from non-engrafted mice lacking human immune cells (NE tumor A and B), two PDX tumors from mice with autologous engraftment (HuMo A and B), and a cell line derived from the patient’s tumor (figure 3D). Five additional changes were shared among the tumor samples and absent from the cell line, with 36 additional mutations being unique to the cell line, and the fewest unique mutations in the humanized mouse specimens (15 and 16 mutations in HuMo A and B, respectively). These data underscore the capacity of the autologous PDX method to recapitulate the somatic heterogeneity that the patient tumor encompasses.

### Autologous MISTRG6 mice display diverse human immune cell populations circulating in the blood and within the tumor, and they recapitulate an immunosuppressive tumor microenvironment.

**Figure 4:**
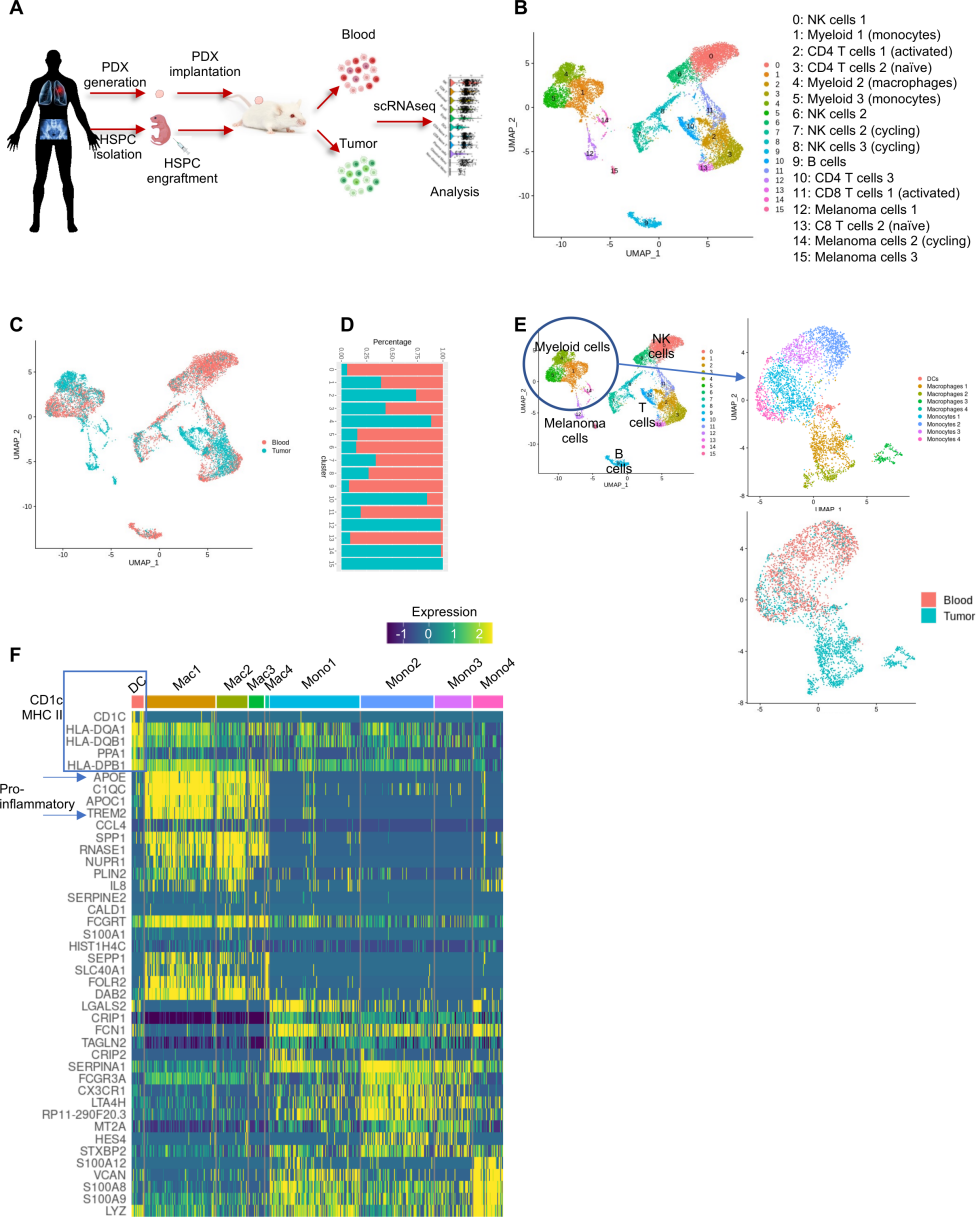
Single cell genomics reveals multiple human immune cell types in tumors and blood of autologous MISTRG6 PDX mice, including innate immune cell types present in the TME. (A) Schematic representation of scRNAseq experiments. (B) UMAP embedding displaying unsupervised clustering of 14,603 human cells from blood and melanoma PDX of autologous MISTRG6 mice. Cell types were identified by marker genes and identities are listed. (C) UMAP embedding displaying tissue of origin for cells in A; red cells are derived from blood libraries, blue cells from tumor libraries. (D) Proportions of cluster representation in blood versus tumor scRNAseq libraries. (E) Re-clustering of myeloid cells reveals substructure of nine clusters including DCs, macrophages and monocytes in differential tissue representation as indicated in the bottom panel. (F) Heatmap indicating expression of top differentially expressed genes between each cluster, highlighting presence of human DCs in TME and pro-inflammatory macrophage subtypes. DCs, dendritic cells; HSPCs, hematopoietic stem and progenitor cells; NK, natural killer; PDX, patient-derived xenograft; scRNAseq, single-cell RNA sequencing; TME, tumor microenvironment; UMAP, Uniform Manifold Approximation and Projection.

To fully characterize the autologous MISTRG6 model and investigate mechanisms by which autologous human immune cells enhance tumor growth, we performed single cell transcriptomics on hCD45^+^-enriched cells from blood and tumor isolated from Mel1199 and Mel1824 mice (figure 4A). After filtering for bona fide human cells of high quality, 9044 cells from blood and 5559 cells from tumor were analyzed from the two autologous models. All cells were combined, clustered and visualized using Uniform Manifold Approximation and Projection (UMAP). Examination of cluster-specific gene markers revealed 16 distinct cell subtypes, including 3 myeloids, 2 NK cells, 2 CD8 T cells, 3 CD4 T cells, 2 cycling lymphocytes, 1 B cell, and 3 melanoma cell clusters (figure 4B). As expected, these subtypes were differentially represented across tissues (tumor vs blood). For example, monocyte cluster 1 was more highly enriched in blood, while the macrophage cluster was dominant in the TME (figure 4C, D).

Given the importance of myeloid cell subtypes in promoting and inhibiting tumor growth, and the unique presence and functionality of these human cells in autologous MISTRG6 models, we reclustered the data to reveal the myeloid subtypes present (figure 4E). This revealed nine distinct clusters including four monocyte (distinguished by genes such as LGALS2, SERPINA1, FCGR3A, LYZ), four macrophage (defined by APOE, C1QC, APOC1, TREM2), and 1 DC (with high expression of CD1C, FCER1A, FLT3, MHC II genes) clusters (figure 4F). As expected, the majority of DC and macrophage cells were detected in the tumor, while most monocytes were present in the blood, reflecting their expected biological homing and plasticity (figure 4C–E).

**Figure 5:**
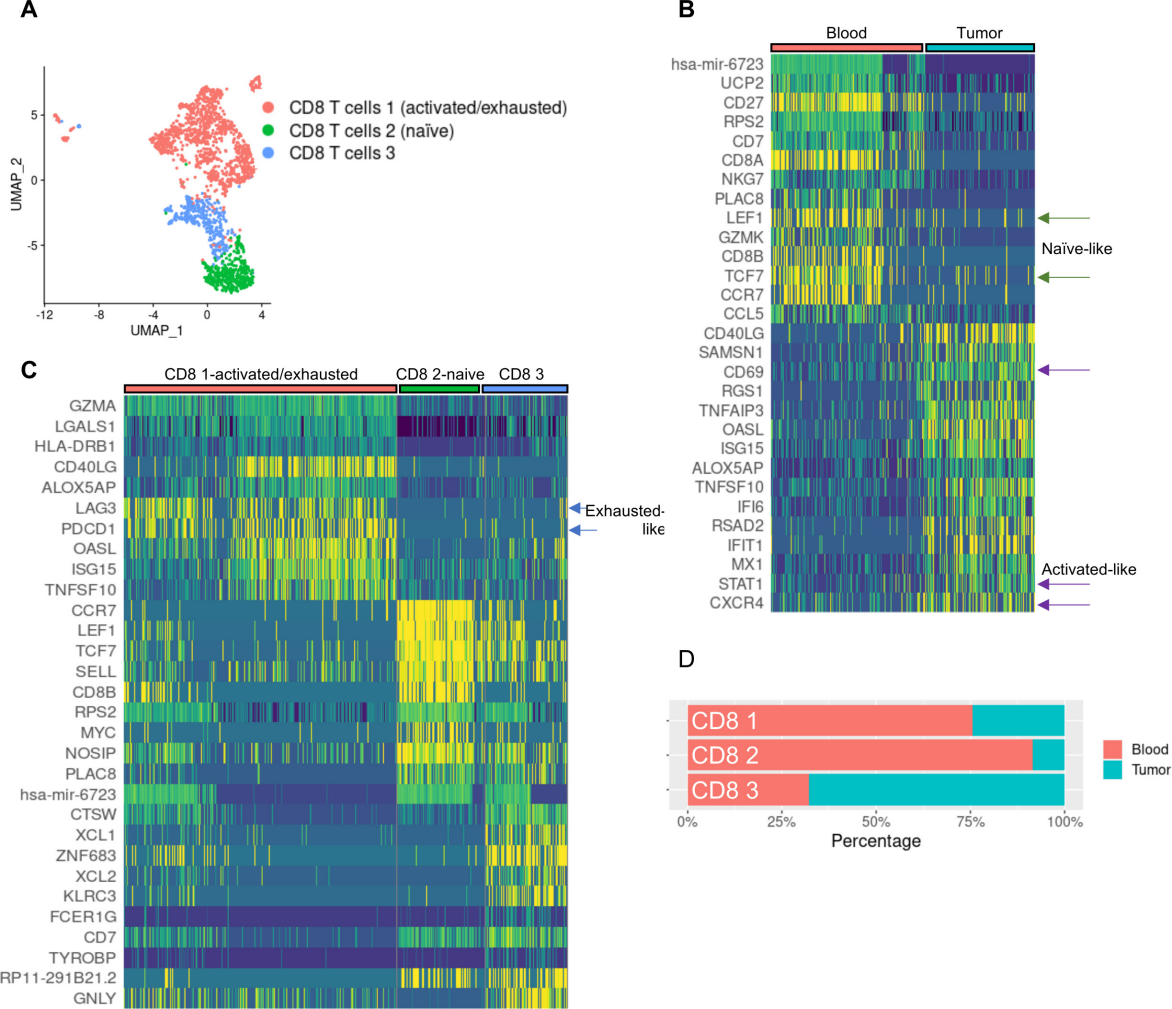
CD8 T cells circulating in the blood of autologous MISTRG6 mice display features of naïve states while those in the TME express markers of activation and exhaustion. (A) Re-clustering of CD8 T cells reveals substructure of three clusters. (B) Differentially-expressed genes between CD8 T cells in blood versus melanoma PDX display features of naïve (blood) and activated (tumor) states. (C) Heatmap indicating expression of top differentially expressed genes between each cluster, highlighting activated/exhausted-like phenotype of CD8 1 cluster. (D) Cluster representation of CD8 T cell subclusters in tissues, demonstrating over-representation of activated/exhausted-like CD8 T cells in the tumor microenvironment of autologous mice. PDX, patient-derived xenograft; TME, tumor microenvironment; UMAP, Uniform Manifold Approximation and Projection.

Given the central importance of CD8 T cells as effectors of antitumor immune responses, we next characterized the CD8 T cell compartments in autologous models by performing focused subclustering of human CD8 T cells (figure 5A). Comparing CD8 T cells present in blood versus tumor revealed that the most differentially expressed genes (DEGs) found in blood were characteristic of naïve T cells (eg, LEF1, TCF7, SELL), while genes present in the TME were consistent with activated T-cell phenotypes, such as CD69, STAT1, and CXCR4 (figure 5B). In addition, subclustering revealed three distinct CD8 T cell types that included two activated-like populations expressing genes such as CD40LG, XCL1, and XCL2, with one of these populations also expressing an activated/exhausted program typified by expression of PDCD1, LAG3, and GZMA. The third CD8 population expressed naïve-type genes such as LEF1, TCF7, SELL (figure 5C). Both naïve-like and activated/exhausted-like genes were present in the blood and TME, suggesting that human T cells may become activated in the TME or in other settings in the body (figure 5D).

CD4 T cells subclustered into five distinct groups with divergent localizations between blood and tumor; clusters 1, 3 and 5 were more prevalent in blood, while clusters 2 and 4 were almost exclusively present in tumor (online supplemental figure 3A-C). Cluster 1 was characterized by naïve T-cell genes with a subset of FOXP3-expressing T regulatory cells, while the TME-associated cluster 4 displayed high expression of interferon-signature genes (eg, IFIT1, IFIT2, IFIT3, MX1), suggesting that these may facilitate effector T-cell function (online supplemental figure 3C, D).

**Figure 6:**
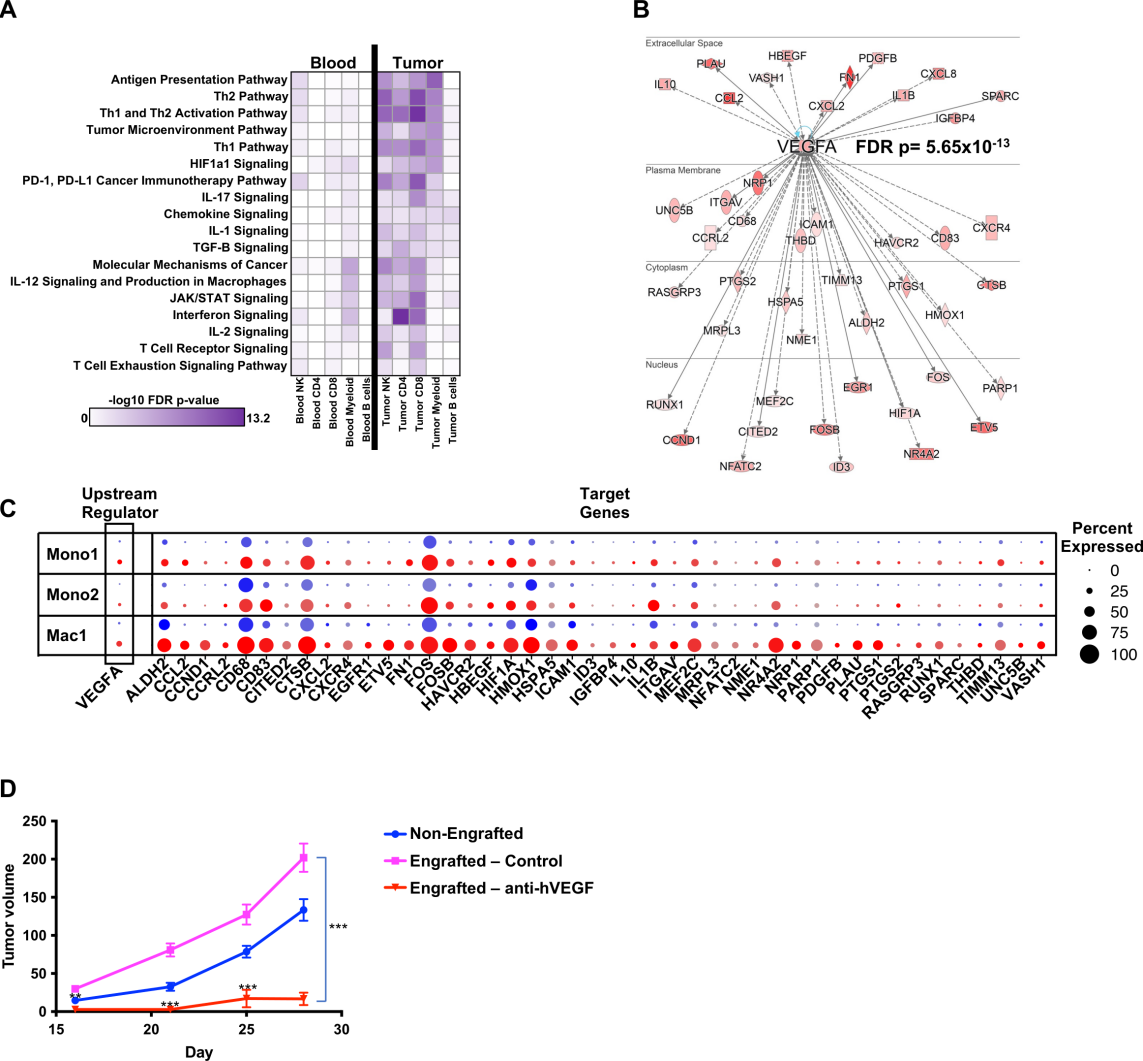
Immune cells in the TME display gene signatures associated with immune activation and signaling, including VEGF-A signaling, and blockade of this molecule abrogates enhanced tumor growth in engrafted autologous PDX mice. (A) Canonical pathway representation in indicated cell types from blood (left) and melanoma tumor PDX (right). (B) Upstream pathway analysis identifies VEGFA and its target genes as highly represented in the TME; red shading indicates VEGFA target gene expression level in the TME. (C) VEGFA and its target genes are over-represented in the TME (red) of myeloid gene clusters (monocyte 1, monocyte 2, macrophage 1) compared with blood (blue); size of split dot plot circle indicates percent expression of each gene among cells of that cluster, while intensity of color indicates level of expression. (D) Treatment of autologously engrafted MISTRG6 Mel2 PDX mice with bevacizumab, a clinical anti-hVEGF-A therapy, significantly reduces tumor growth compared with autologous HSPC-engrafted control (asterisks indicate comparison between drug-treated and control engrafted mice); n=13 non-engrafted, n=4 engrafted control, n=5 bevacizumab-treated engrafted mice. FDR, false discovery rate; HSPC, hematopoietic stem and progenitor cell; PDX, patient-derived xenograft; TME, tumor microenvironment.

To further define the genes and pathways dominant in the autologous MISTRG6 model, we used Ingenuity Pathway Analysis (IPA) to analyze pathways over-represented in tumors compared with blood across cell types. We found significant associations with T-cell activation in tumors including T cell receptor signaling, Th1, Th2, interferon signaling, Jak/STAT signaling, IL-17 signaling, antigen presentation pathways (figure 6A). In addition, genes representing the programmed cell death-1 and programmed cell death ligand-1 (PD-1/PD-L1) cancer immunotherapy, T cell exhaustion signaling, and tumor microenvironment pathways were significantly associated with the TME.

Notably, IPA Upstream Regulator Analysis identified VEGFA, a central player in tumor growth and vascularization across multiple tumor types,^18–20^ as a key upstream inducer of genes in the TME (false discovery rate p=5.65×10^−13^, figure 6B). Indeed, expression VEGFA itself was nearly absent in blood but induced in the TME, especially in macrophages (figure 6C). In addition, VEGFA direct and indirect targets were highly represented among the DEGs between tumor and blood in the myeloid cell types (figure 6B, C).

### Inhibiting the actions of human VEGF-A blocks the enhanced tumor growth in autologously engrafted mice

To test the imputed relevance of VEGF-A in the TME and its effect on tumor growth, we chose to selectively block human VEGF-A as the murine subcutaneous microenvironment is replete with murine VEGF which could compound the results. Thus, we treated autologous mice humanized with HSPCs and PDXs from Mel2 with the anti-human-VEGF-A antibody bevacizumab that has high affinity for human VEGF-A yet low affinity for mouse VEGF-A and which does not affect tumor growth in non-engrafted hosts.^10 21^ The Mel2 PDXs grown in autologously engrafted MISTRG6 mice grew significantly larger than those in non-engrafted littermate control hosts. When treated with bevacizumab (10 mg/kg, weekly intraperitoneal injection), the enhanced tumor growth seen with human autologous engraftment was significantly abrogated, with bevacizumab-treated mice bearing significantly smaller tumors compared with controls (figure 6D). Thus, these *in silico* and *in vivo* results suggest that human VEGF-A production in the TME of mice bearing an autologous human TME enhances tumor growth in MISTRG6 PDX models.

## Discussion

While advances in immunotherapy have greatly improved quality and length of life for people with cancer, there remains significant variability in individual patient responses to treatment. For example, nearly 40% of patients with metastatic melanoma, a disease with among the most immunotherapy successes, do not respond initially to combination cytotoxic T lymphocyte associated protein 4(CTLA-4) and PD-1 inhibition and of those that do respond, the duration of response is limited by as-yet undefined mechanisms of acquired resistance.^22 23^ Hence, there is a need for personalized models that faithfully reflect the TME of individual patients, and that can therefore be used to test primary and acquired resistance to therapies. By engrafting mice with BM derived stem cells followed by implantation of tumor derived from the same donor, we have demonstrated that autologous MISTRG6 models recapitulate important features of the human TME, including sufficient immunosuppression to prevent tumor clearance, presence of activated/exhausted-like T cells, and harboring innate immune cells including DCs, monocytes, NK cells, and macrophages, the latter being especially relevant by the production of VEGF-A.

While humanized mice have long been used for engraftment of human hematopoietic cells to study myriad processes,^24^ MISTRG6 mice engrafted with relatively few HSPCs develop a humanized TME that reflects the immune makeup of living patients. Indeed, we found that a low-volume BM aspiration can provide sufficient HSPCs (1–5 × 10^6^) to engraft multiple mice on a scale not seen previously. These cohorts are large enough to facilitate drug or other comparisons, opening the door to exciting experiments such as prospective co-clinical trials.

Importantly, MISTRG6 mice engrafted autologously with BM-derived patient HSPCs support tumor growth for multiple weeks (typically 5–7 weeks post tumor placement), permitting study of tumor expansion in a timeline similar to other models. We have noted metastatic lesions in multiple organs of autologously-engrafted MISTRG6 mice (not shown). Notably, we found that tumors grown in autologously-engrafted hosts were larger than those grown in non-HSPC-engrafted hosts, underscoring the functionality of the human cell engraftment and the pro-growth effects that the TME can acquire and provide. However, not all autologous models displayed enhanced growth in the presence of autologous human cells, underlining the inter-patient differences that our MISTRG6 model elucidates and that will be important for future personalized medicine approaches.

The ability to study the myeloid cell fraction of the TME is critical to understanding tumor-immune interactions. Indeed, myeloid lineage cells, such as macrophages and dendritic cells, are major components of a patient’s TME that are recapitulated in our approach, and these innate immune cells have drastic and varied effects on tumor growth as well as immune function^25 26^ Our single cell transcriptomic analyses pointed to the myeloid compartment as producers of VEGF-A in the TME, a central protumor cytokine. In support of this cytokine’s importance, functional inhibition of human VEGF-A with bevacizumab abrogated the enhanced growth effects in the model and indicates the potential of the model for preclinical drug testing.

Myeloid cells are also important orchestrators of adaptive immune responses, functioning as antigen-capturing and antigen-presenting cells, as well as modulators of the activity of other immune cells, especially effector T cells. Each of these cell types can have direct protumor and antitumor effects on malignant cells. For example, macrophages can exhibit pro-inflammatory or anti-inflammatory phenotypes. The former are linked with type 1 inflammation, intracellular pathogen killing, and tumor resistance, while the latter exhibit immunoregulatory and pro-angiogenic properties, promote tissue repair, and contribute to tumor progression.^3 27–30^ In this regard, the demonstration of several macrophage subtypes in the TME of autologously engrafted MISTRG6 mice may provide a way to decipher these processes *in vivo*. While absence of human thymus in this system suggests that T cells are selected in mouse thymus, our data show that a subset of CD8 T cells are activated, including in the TME. The presence of DCs in the model is significant given the importance of these antigen presenting cells in tumor immune responses, and further investigations in the model may elucidate further mechanisms of CD8 activation including how the T cells in humanized mice interact with human MHC on DCs. In addition, the specific epitopes recognized, as well as the possibility of bystander T-cell activation, remain active areas of investigation to explain CD8 T cell activation in the blood and TME.

Thus, due to their unique sensing abilities, their functional plasticity, and their central position in the TME, macrophages, monocytes, and DCs are essential elements to investigate the variability in response to cancer and to its treatment. It is critically important to credential a model of humanized mice where these lineages are properly developed and functionally mature.

Like other preclinical models, our method has limitations. While MISTRG6 mice bearing autologous tumor and TME elements develop major innate immune cell types as discussed, like most other humanized mouse models, they lack large numbers of human neutrophils, basophils, and eosinophils; further research is required to identify the genes required to support the development of these human cell types. In addition, like other PDX methods, the autologous technique is limited by the fact that not all fresh tumor tissue samples generate efficient PDXs that grow robustly in the timeline of an experiment, and, while PDXs recapitulate the heterogeneity and behavior of tumors better than cell lines adapted for growth *in vitro*, they require selection in non-engrafted hosts which may select for more aggressive phenotypes.^31–33^ In addition, the yield of BM aspiration is variable, and in rare cases too few HSPCs are obtained. Further humanization of recipient mice will likely improve the ability to engraft these cell types, such that autologous mice may be engrafted with fewer and fewer adult hematopoietic stem cells. Finally, the chronological lag between tissue collection and autologous modeling inherent in the technique due to the need for PDX generation, which can take weeks or months, may limit the ability to use the model as a predictor of primary therapies. However, given that surgical tissue is often collected early in a patient’s clinical course (eg, for resection of a primary NSCLC), autologous modeling can begin early in a patient’s course, and bear fruit in modeling second or third therapies. Furthermore, PDX models can be successfully generated from small biopsy specimens, broadening the applicability to most metastatic solid tumors.^31 34 35^ Our findings demonstrate the broad utility of a genetically matched, fully autologous humanized mouse model system for investigating the TME and treatment responses of individual patients with solid tumor.

## Methods

### Mice

Mouse studies were performed under protocols approved by the Yale Office of Animal Research Support and Institutional Animal Care & Use Committee 2019–11396. MISTRG6 mice bear human knock-ins of M-CSF (CSF1), CSF2/IL3, SIRPA, THPO genes were humanized on a RAG2^−/−^, IL2RG^−/−^ mixed BALB/c-129 background, as previously described.^10 13 14^ Mice were maintained in a BSL-2 mouse facility on a 14–10 hour light-dark cycle with 2 weeks on, 2 weeks off sulfamethoxazole/trimethoprim diet. Experimental mice were cross-fostered with CD1 female mice from Charles River Laboratories for maintenance of eubiotic flora. Newborn mice engrafted with adult HSPCs were preconditioned with 150 rads of irradiation using an X-ray irradiator prior to intrahepatic injection as previously described.^10^ Sample sizes for autologous experiments were determined by availability of patient HSPCs. Mice were excluded from analyses if human hematopoietic engraftment rate was less than 5%. For drug experiments, mice were randomized based on %hCD45+ prior to therapy initiation. All mice were co-housed within experiments where appropriate. Investigators were blinded at group allocation.

### Human HSPC isolation and enrichment

All human samples were obtained and handled in accordance with established Yale and Washington University Human Investigational Committee protocols. Candidates for participation were identified by investigators, and subsequently approached by investigators or clinical trials staff to discuss participation in this study. FL HSPCs were purified as previously described.^36^ BM aspirates were performed using a 15 gauge Illinois needle (VWR) with standard technique. Aspiration of 40–150 cc of BM from unilateral or bilateral iliac crests was done. Peripheral blood was also collected from an intravenous line placed in the normal course of perioperative care. The BM aspirate, peripheral blood, and a component of the resected tumor were subsequently transported to our laboratory for further processing. Freshly-aspirated BM and peripheral blood were anticoagulated with sterile EDTA and subjected to Ficoll density-mediated separation for isolation of mononuclear cells. CD34+ cells were positively selected using the EasySep Human CD34 Positive Selection Kit (STEMCELL Technologies) according to the manufacturer’s protocol. HSPCs and PBMCs were cryopreserved in 90% fetal bovine serum (FBC) + 10% dimethylsulfoxide (DMSO) until use.

### Tissue processing and flow cytometry

Fresh excess human tumor tissue was obtained from enrolled patients and divided into 1–2 mm^3^ pieces for subcutaneous implantation in the flank of MISTRG6 or NSG mice lacking human HSPC reconstitution, as previously described.^34 37^ Tumor growth was monitored by caliper measurements, and when tumors reached 500–1000 mm^3^, mice were euthanized and tumor tissue again divided into 1–2 mm^3^ pieces for PDX passaging. PDXs were passaged two to three times, and histology confirmed with H&E staining. PDXs were cryopreserved in 90% FBS+10% DMSO in liquid nitrogen until use.

Tumors, spleen, lung, and liver tissues were minced in phosphate-buffered saline (PBS)+1% FBS and digested with collagenase D (1 mg/mL, Sigma) and DNAse at 37°C for 15 min. Tumor-infiltrating immune cells were isolated with a 40–80% Percoll density gradient (Sigma). Non-specific staining was blocked with human (BD Biosciences) and mouse (Bio X Cell, BE0307) Fc block for 10 min. Epitopes were stained at 4°C for 30 minutes and cells were fixed with 4% paraformaldehyde; intracellular staining was performed using BD permeabilization buffer (BD Biosciences). Samples were analyzed on an LSRII flow cytometer, and data parsed with FlowJo software V.10 as well as GraphPad Prism V.9. Cell sorting was performed using a BD FACSAria flow sorter under BSL2 containment. Sorted cells were subjected to 10X Genomics 5’V2 library construction and sequenced using NovaSeq. Data were filtered as described previously.^36^

### Immunofluorescence staining

Cryosections (8 um) were acetone fixed, air dried, washed with PBS and consecutively treated with Fc Receptor Block (Innovex Bioscience) for 40 min+Background Buster (Innovex Biosciences) for an additional 30 min. The sections were then stained with directly conjugated antibody mix in PBS 5% bovine serum albumin (BSA) 0.1% Saponin for 1 hour at room temperature and washed. Nuclei were counterstained with SytoxBlue 1:1000 for 2 min. Tissues were mounted in Fluoromount-G mounting media. Images were acquired using a Leica SP8 confocal microscope.

### Statistical analyses

Unpaired parametric t-tests were used to compare immune cell frequencies and tumor sizes, except where indicated otherwise in figure legends. Bars indicate mean±SEM., except where indicated otherwise. The single-cell RNA sequencing data analysis was performed using Seurat V.4.0.1 R package,^38^ including cell type stratification and comparative analyses between tumor and blood samples. In the quality control analysis, poor-quality cells with <250 (likely cell fragments) or >5000 (potentially doublets) unique expressed genes were excluded. Cells were removed if their mitochondrial gene percentages were over 25% or if their ratios of reads mapped to the human genome (over the total reads mapped to the humanized mouse genome) were lower than 90%. The data was first integrated with default settings in Seurat (using 30 dimensions in the anchor weighting procedure), followed by principal component analysis for dimensionality reduction. We retained 30 leading principal components for further visualization and cell clustering. The UMAP algorithm was used to visualize cells on a two-dimensional space.^39^ Subsequently, the share nearest neighbor graph was constructed by calculating the Jaccard index between each cell and its 20-nearest neighbors, which was then used for cell clustering based on Louvain algorithm (with a resolution of 0.5). Each cluster was screened for marker genes by differential expression analysis based on the non-parametric Wilcoxon rank-sum test. Based on checking the expression profile of those cluster-specific markers, we identified 16 distinct cell types, including different subtypes of CD4/CD8 T cells, B cells, myeloid cells, NK cells, and melanoma cells. In the downstream analysis, we focused on myeloid cells, CD8 T cells, and CD4 T cells, respectively. Specifically, we separated out each cell type and repeated the clustering to define cell subtypes (states) in a high-resolution manner. Differential expression analysis was performed to (1) reveal the functional role of each subtype, and (2) identify DEGs between the blood and tumor conditions. Top representative DEGs were visualized using heatmaps or dot plots.

For Ingenuity Pathway Analysis, differentially expressed genes were identified by filtering group comparisons for biological (ABS(FC) > 1.5) and statistical significance (adjusted p<0.05). These were uploaded into IPA software (QIAGEN, Content V.73620684, 2022 http://www.ingenuity.com), mapped to their corresponding IPA identifier, and used for functional analysis. Pathways and upstream regulators were determined using an FDR p<0.05 calculated with a right-tailed Fisher’s exact test.

### Sequence alignment

WES data were processed using the immuno.cwl analysis workflow (https://github.com/genome/analysis-workflows/tree/master/definitions/pipelines). Briefly all alignment data was processed through xenome (1.0.0) and reads classified as either ‘graft’ (*H. sapiens*) or ‘both’ were retained in order to remove incidental data from the host xenograft samples. WES data was then aligned to GRCH38 via bwa-mem (0.7.15) and read duplicates marked with picard (2.18.1). base quality score recalibration (BQSR) correction was performed via GATK (4.1.8.1). Variant calling was performed via Mutect (GATK 4.2.3.0), Strelka (2.9.9), VarScan 2 (2.4.2), and Pindel (1.4.2), and a log-likelihood ratio filter applied. Called variants were further refined by the following criteria: variant caller count >2, tumor depth ≥50 and tumor variant count ≥2, normal depth ≥20 and normal variant count ≤3. Variant intersection among related samples was then performed using R (4.1.1) and Upset plots created using the UpSetR R package.

### Antibodies

mCD45 (Clone: 30-F11), hCD45 (HI30), hCD3 (UCHT1), hCD14 (HCD14), hCD16 (3G8), hCD19 (HIB19), hCD33 (WM53), hCD20 (2H7), hCD11B (M1/70), hCD11C (3.9), HLA-DR (LN3), hPD1 (A17188B), hNKp46 (9E2), hCD4 (OKT4), hCD8 (SK1), mCD117 (2B8), hCD117 (104D2), mSca1 (D7), hCD34 (581), hCD38 (HIT2), mCD34 (HM34), mCD38 (90), hCD31 (WM59). All antibodies were obtained from BioLegend and used at 1:300 dilution in PBS+1% fetal calf serum (FCS).

## Data Availability

Data are available upon reasonable request.
