# Supplementary material for: Autologous humanized PDX modeling for immuno-oncology recapitulates features of the human tumor microenvironment
Source: J Immunother Cancer. 2023 Jul 24;11(7):e006921. doi: 10.1136/jitc-2023-006921 (PMC10373695; doi:10.1136/jitc-2023-006921)
Supplement: Supplementary data [file jitc-2023-006921supp001.pdf]

Supplementary Figure 1

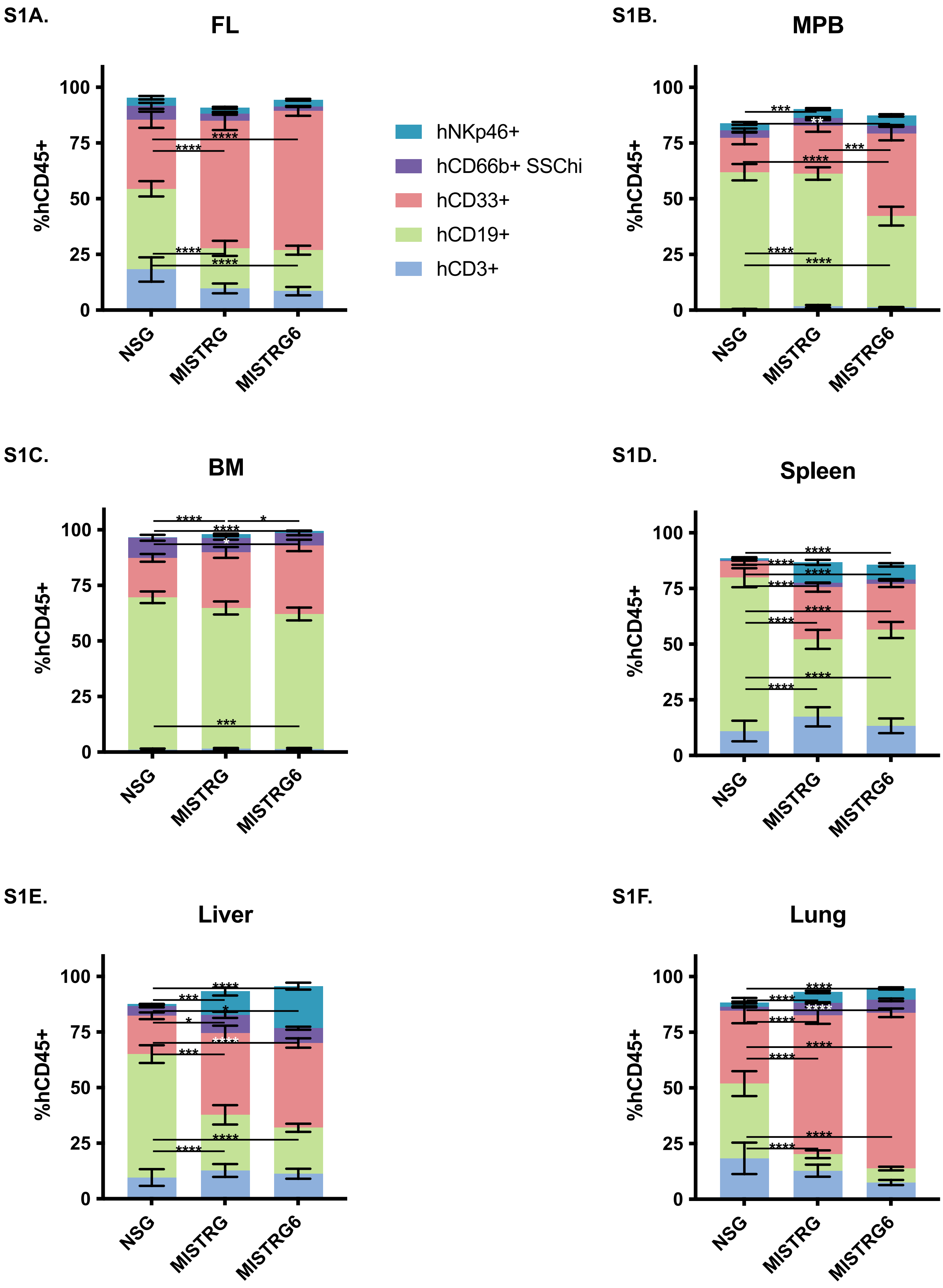

Supplementary Figure 2

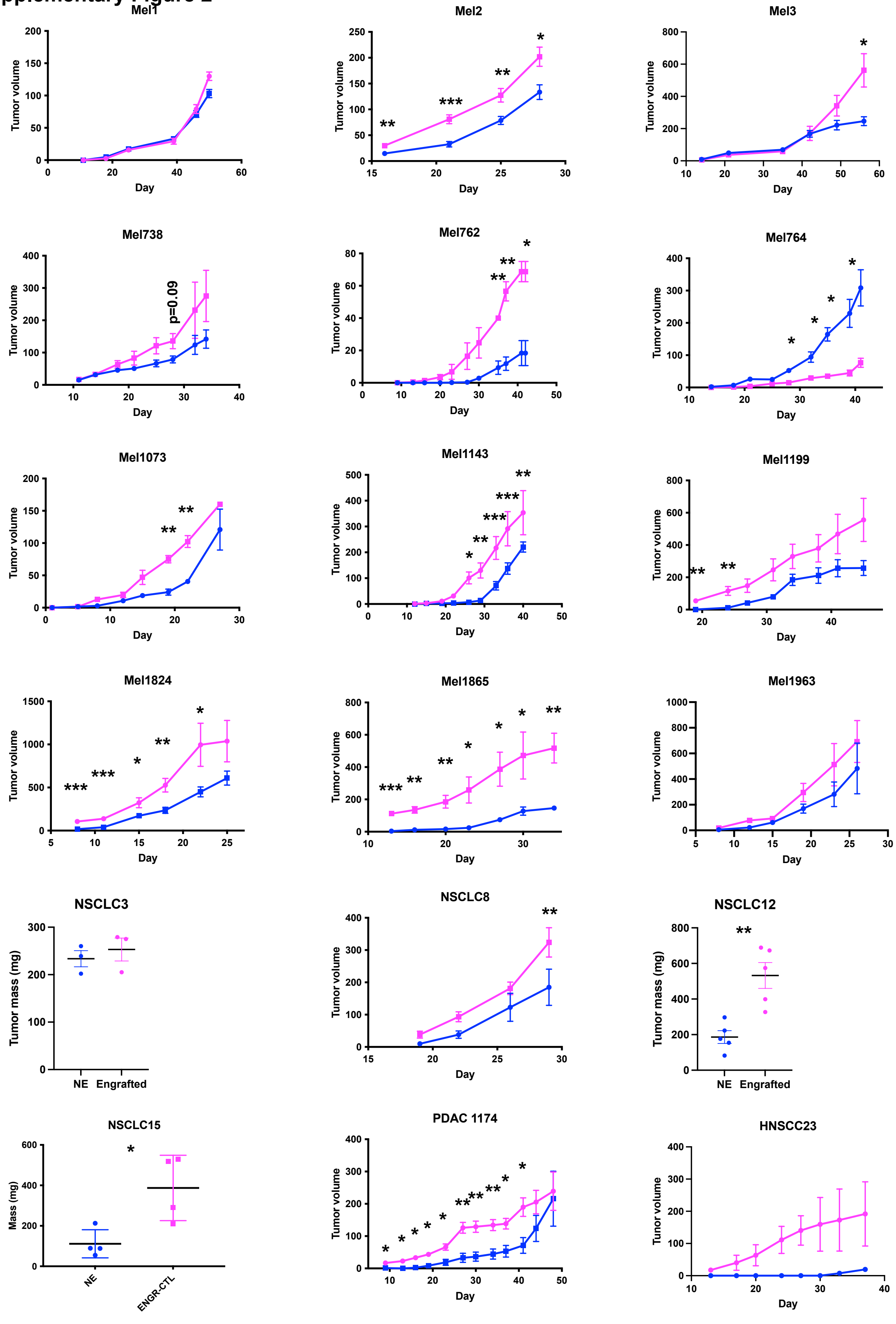

Supplementary Figure 3

S3A.

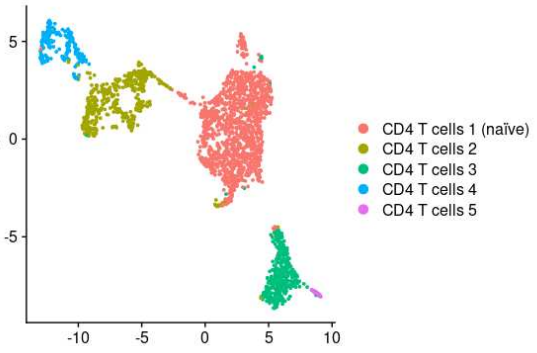

S3B.

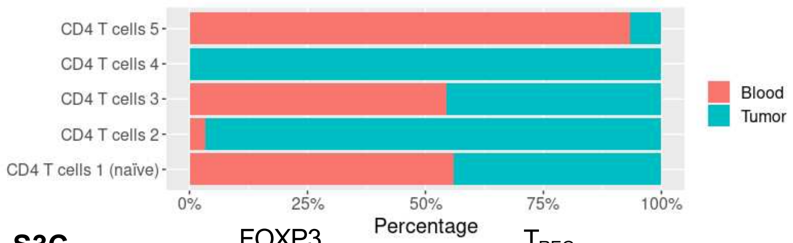

S3C.

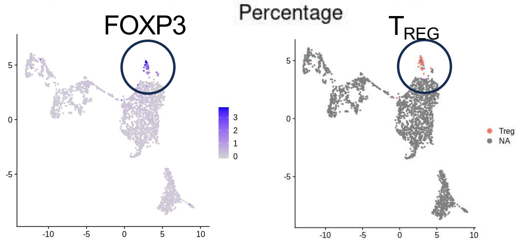

S3D.

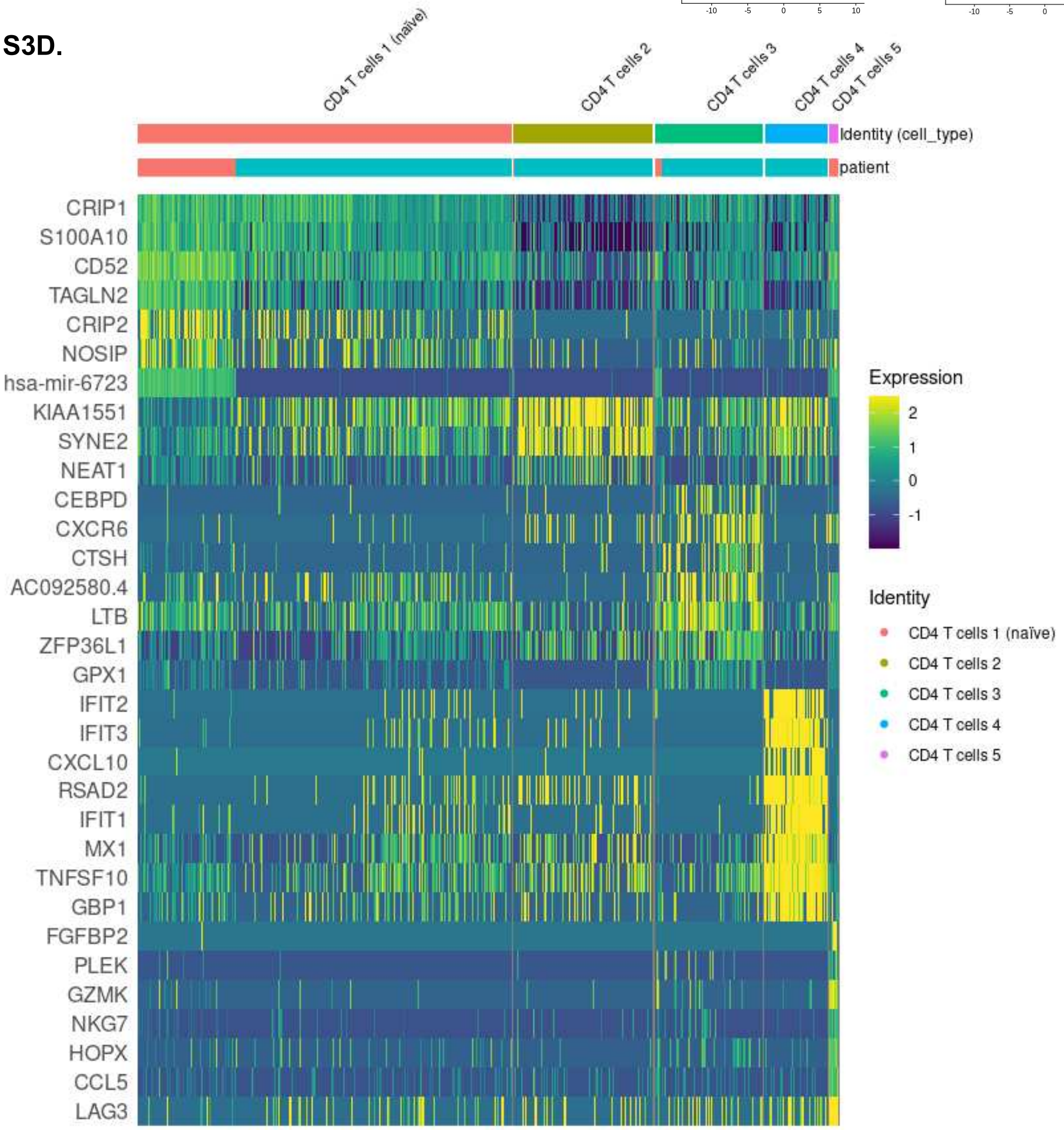

Supplementary Table 1

Patient	Age	Sex	Tumor Type	Diagnosis	CD34+ cell #	PDX?
1	65	F	NSCLC	Stage IA adenocarcinoma	<100k	
2	73	F	NSCLC	Stage IA adenocarcinoma	150k	
3	82	F	NSCLC	Stage IA squamous cell carcinoma	3.9 million	Yes
4	56	M	NSCLC	Stage IIB large cell carcinoma	4 million	
5	70	F	NSCLC	Mucoepidermoid carcinoma	5.1 million	
6	57	F	NSCLC	Adenosquamous carcinoma	7 million	
7	70	M	NSCLC	Stage IB adenocarcinoma	6.5 million	Yes
8	62	F	NSCLC	Stage IA adenocarcinoma	6 million	Yes
9	79	M	NSCLC	Stage IIA squamous cell carcinoma	3.7 million	Yes
10	71	M	NSCLC	Stage IA adenocarcinoma	14 million	
11	63	F	NSCLC	Stage IA adenocarcinoma	8.2 million	
12	79	F	NSCLC	Stage IV squamous cell carcinoma	2.7 million	Yes
13	54	F	NSCLC	Stage II adenocarcinoma	5.0 million	
14	57	M	NSCLC	Stage IV NSCLC	2.5 million	
15	72	M	NSCLC	Stage IV adenocarcinoma	3.6 million	Yes
16	74	M	NSCLC	Stage IV adenocarcinoma	3.4 million	
17	73	M	NSCLC	Stage IV neuroendocrine tumor	6.6 million	
18	72	F	NSCLC	Stage IV NSCLC	4 million	
19	79	F	NSCLC	Stage IV NSCLC	2.7 million	Yes
20	78	F	HNSCC	Stage II HNSCC of lateral tongue	1 million	Yes
21	63	F	HNSCC	Stage IVa HNSCC of buccal mucosa	2 million	Yes
22	46	F	Melanoma	Stage IV melanoma	6 million	
23	70	F	Melanoma	Stage IIIc melanoma	250k	
24	62	M	Melanoma	Stage IV melanoma	2.2 million	
25	67	M	Melanoma	Melanoma	1.8 million	
26	61	M	Melanoma	Stage IIIc melanoma	100k	
27	34	M	Melanoma	Melanoma	1.9 million	
28	61	F	Melanoma	Stage IV melanoma	1 million	
29	57	M	Melanoma	Stage IV melanoma	2.5 million	Yes
30	55	F	Melanoma	Melanoma	2 million	
31	42	M	Melanoma	Stage IV melanoma	4.8 million	Yes
32	22	M	Melanoma	Stage IIIc melanoma	3 million	
33	52	F	Melanoma	Stage IIIb melanoma	4 million	Yes
34	65	M	Melanoma	Stage IV melanoma	n/a	
35	71	F	Melanoma	Melanoma	500k	
36	53	M	Melanoma	Stage IIIc melanoma	600k	
37	71	M	Melanoma	Melanoma	80k	
38	65	M	Melanoma	Stage IIIc melanoma	1.5 million	
39	73	M	Melanoma	Stage IIIb melanoma	3.4 million	
40	65	M	Melanoma	Stage IIIb melanoma	1.8 million	
41	43	F	Melanoma	Stage IIIc melanoma	3.6 million	
42	50	F	Melanoma	Stage IIIc melanoma	7 million	
43	42	M	Melanoma	Stage IIIc melanoma	3.6 million	Yes
44	54	F	PDAC	Pancreatic adenocarcinoma	1.2 million	
45	48	M	PDAC	Pancreatic adenocarcinoma	2.5 million	Yes
46	48	M	Melanoma	Stage IV melanoma	2.1 million	Yes
47	67	M	PDAC	Pancreatic adenocarcinoma	1.6 million	Yes
48	85	F	PDAC	Pancreatic adenocarcinoma	6.8 million	Yes
49	80	M	Melanoma	Stage IIIc melanoma	1.95 million	Yes
50	75	F	Melanoma	Stage IIIb melanoma	5.5 million	
51	51	M	Melanoma	Stage IIIc melanoma	8.5 million	Yes
52	74	M	Melanoma	Stage IV melanoma	5 million	Yes
53	35	M	Melanoma	Stage IIIc melanoma	6.1 million	
54	81	M	Melanoma	Stage IIIb melanoma	3 million	
55	63	M	Melanoma	Stage IIIc melanoma	3.1 million	Yes
56	35	M	Melanoma	Stage IIId melanoma	3 million	
57	60	M	Melanoma	Stage IV melanoma	1.4 million	
58	64	F	Melanoma	Stage IV melanoma	2.5 million	
59	57	M	Melanoma	Stage IIIc melanoma	4.5 million	
60	51	F	Melanoma	Stage IIIc melanoma	2.6 million	
61	58	F	Melanoma	Stage IV melanoma	2.4 million	
62	48	M	Melanoma	Stage IIIc melanoma	1.76 million	Yes
63	68	M	Melanoma	Stage IV melanoma	4 million	Yes
64	30	M	Melanoma	Stage IIIc melanoma	12 million	
65	79	M	Melanoma	Stage III melanoma	2.8 million	Yes
66	47	M	Melanoma	Stage IIIc melanoma	2.4 million	
67	42	M	Melanoma	Stage IIIb melanoma	4.6 million	Yes
68	83	M	Melanoma	Stage IIId melanoma	2.8 million	
69	33	M	Melanoma	Stage IIId melanoma	4.8 million	
70	58	M	Melanoma	Stage IIIc melanoma	1.8 million	
71	57	F	Melanoma	Stage IIIc melanoma	820k	

Supplementary Figure Legends:

Supplementary Figure 1: MISTRG6 mice display robust peripheral blood and tissue engraftment of human cell types, including innate immune cells.
A. Human hematopoietic cells in peripheral blood from mice of indicated strains engrafted with FL-derived $CD34^+$ cells, including T cells ($hCD3^+$, blue), B cells ($hCD19^+$, green), myeloid cells ($hCD33^+$, red), neutrophils (hCD66b, SSC^{hi}, purple), NK cells ($hNKp46^+$, teal).
B. Human hematopoietic cell profiles in peripheral blood from mice of indicated strains engrafted with $CD34^+$ cells isolated from mobilized peripheral blood (MPB) of adult donors.
C. Human hematopoietic cell profiles in BM from mice of indicated strains engrafted with FL-derived $CD34^+$ cells.
D. Human hematopoietic cell profiles in spleen from mice of indicated strains engrafted with FL-derived $CD34^+$ cells.
E. Human hematopoietic cell profiles in liver from mice of indicated strains engrafted with FL-derived $CD34^+$ cells.
F. Human hematopoietic cell profiles in lung from mice of indicated strains engrafted with FL-derived $CD34^+$ cells.

Supplementary Figure 2: Autologously-engrafted MISTRG6 mice support enhanced tumor growth in multiple patient models.
Tumor growth curves for non-engrafted (blue) and autologously engrafted (magenta) PDX mice representing indicated patients; * $p<0.05$, ** $p<0.01$, *** $p<0.001$, unpaired parametric t-test; bars indicate mean and S.E.M. Mel1 n=3 non-engrafted, n=8 engrafted; Mel2 n=13 non-engrafted, n=4 engrafted; Mel3 n=6 non-engrafted, n=5 engrafted; Mel738 n=3 non-engrafted, n=3 engrafted; Mel762 n=3 non-engrafted, n=3 engrafted; Mel764 n=3 non-engrafted, n=3 engrafted; Mel1073 n=4 non-engrafted, n=5 engrafted; Mel1143 n=5 non-engrafted, n=5 engrafted; Mel1199 n=8 non-engrafted, n=12 engrafted; Mel1824 n=6 non-engrafted, n=5 engrafted; Mel1865 n=4 non-engrafted, n=5 engrafted; Mel1963 n=5 non-engrafted, n=4 engrafted; NSCLC3 n=3 non-engrafted, 3 engrafted; NSCLC8 n=6 non-engrafted, n=6 engrafted; NSCLC12 n=5 non-engrafted, n=5 engrafted; NSCLC15 n=4 non-engrafted, n=4 engrafted; PDAC1174 n=4 non-engrafted, n=9 engrafted; HNSCC23 n=2 non-engrafted, n=3 engrafted.

Supplementary Figure 3: Blood and tumor-resident CD4 T cells display distinct transcriptional states including naïve and interferon-responsive subtypes.
A. Re-clustering of CD4 T cells reveals sub-structure of 5 clusters.
B. Cluster representation of CD4 T cell subclusters in tissues.
C. Expression of FOXP3 gene in a subset of CD4 T cells.
D. Heatmap indicating expression of top differentially expressed genes between each cluster, highlighting interferon activation signature of CD4 4 cluster.

Supplementary Table 1.

Clinical data of patients enrolled in prospective study collecting tumor and BM from solid tumor patients; indicated PDXs grew sufficiently for autologous humanized mouse modeling.
